# It’s Not Just About the Tools: Emotionally Responsive GenAI Education

**DOI:** 10.5334/pme.2280

**Published:** 2026-04-08

**Authors:** Anita Samuel, Jerusalem Merkebu

**Affiliations:** 1 Department of Health Professions Education, School of Medicine, Uniformed Services University of Health Sciences, Bethesda, MD, 20814, US

## Abstract

**Background & Need for Innovation:**

Generative artificial intelligence (GenAI) is rapidly entering health professions education, yet most training emphasizes tool use and ethical guidelines while neglecting emotional readiness. Educators often experience affective discomfort, such as a fear of obsolescence, uncertainty about the ethical use of GenAI, and threats to their professional identity, which can undermine their engagement with GenAI. Few professional development models offer structured support to address these barriers.

**Goal of Innovation:**

To show that emotional readiness is not a secondary concern but the central determinant of GenAI adoption.

**Steps Taken for the Development and Implementation of Innovation:**

We conducted a six-week, fully online, asynchronous course titled AI in Health Professions Education for healthcare professionals that integrated two core innovations: (1) an emotionally responsive, learner-centered environment and (2) a structured assignment framework, FAIR—Familiarization, Application, Interpretation, and Recalibration. The course prioritized emotional safety, instructor vulnerability, and learner agency to support educators through identity disruption and build reflective engagement with GenAI.

**Outcomes of the Innovation:**

Three cohorts (N = 40) participated in the course. Pre-course surveys revealed a mix of experience with GenAI and a wide range of emotional responses, including fear, excitement, and apprehension. Thematic analysis of end-of-course reflections showed a shift from anxiety to confidence and from resistance to leadership. Participants reported using GenAI tools in teaching, curriculum design, and faculty development. Six-month follow-up interviews (n = 5) demonstrated sustained tool use and institutional dissemination. Learners described themselves as change agents and expressed a commitment to supporting peers in the adoption of GenAI.

**Critical Reflection:**

This approach shifted learners from apprehension to leadership, proving emotional safety is key to GenAI adoption. Key pitfalls are the high instructor resource intensity and the entanglement of the FAIR framework and emotional design. We argue that this entanglement is the innovation, and emotional safety is the necessary precondition for the framework’s success.

## Background and Need for Innovation

Medical professionals are under increasing pressure to integrate generative artificial intelligence (GenAI) into their workflows. Most training and support efforts focus on GenAI’s technical capabilities and curricular integration [1]. GenAI represents emergent, unfamiliar, or distrusted technology, and individuals navigating these transitions often experience fear, anxiety, self-doubt, and threats to their professional identity and credibility [2]. While the affective dimension can fundamentally impede readiness for change, it remains under-addressed [3].

Healthcare professionals experience affective discomfort, including fear of obsolescence, uncertainty about the ethical use of AI, and feelings of professional displacement [2,4]. These affective factors shape how educators engage with innovation. However, current training frameworks often overlook how affective discomfort impairs cognitive openness, inhibits identity engagement, and stifles pedagogical or clinical innovation [5]. Consequently, few initiatives provide structured strategies to mitigate these emotional barriers [6]. Failure to address these emotional elements risks entrenching resistance and undermining innovation in healthcare professions.

## Goal of the Innovation

While numerous professional development modules and courses on GenAI for healthcare professionals exist, the vast majority focus on technical proficiency, tool use, and ethical guidelines. They are designed to address a knowledge gap. However, these models often neglect the affective gap. They do not provide structured support for the fear, identity disruption, and anxiety that are associated with the emergence of AI. Our approach posits that emotional readiness is not a secondary concern but the central determinant of GenAI adoption. We designed a process focused first on transforming anxiety into agency, creating a foundation of psychological safety before developing technical skills.

This paper illustrates how a learner-centered, emotionally responsive training development model, with a structured framework, can support healthcare professionals’ engagement with GenAI. We show how intentionally designing for affective engagement can transform anxiety into agency and resistance into reflective innovation. This approach acknowledges and addresses the complex emotional needs inherent in this significant technological disruption.

## Steps Taken for the Development and Implementation of Innovation

We developed a six-week, fully online, asynchronous course titled *“AI in Health Professions Education*” for healthcare professionals. This was a learner-centered, emotionally-responsive course designed around our FAIR framework – Familiarization, Application, Interpretation, and Recalibration. Next, we describe our FAIR framework, followed by an explanation of the emotionally responsive course design.

### FAIR Framework

**Figure 1:**
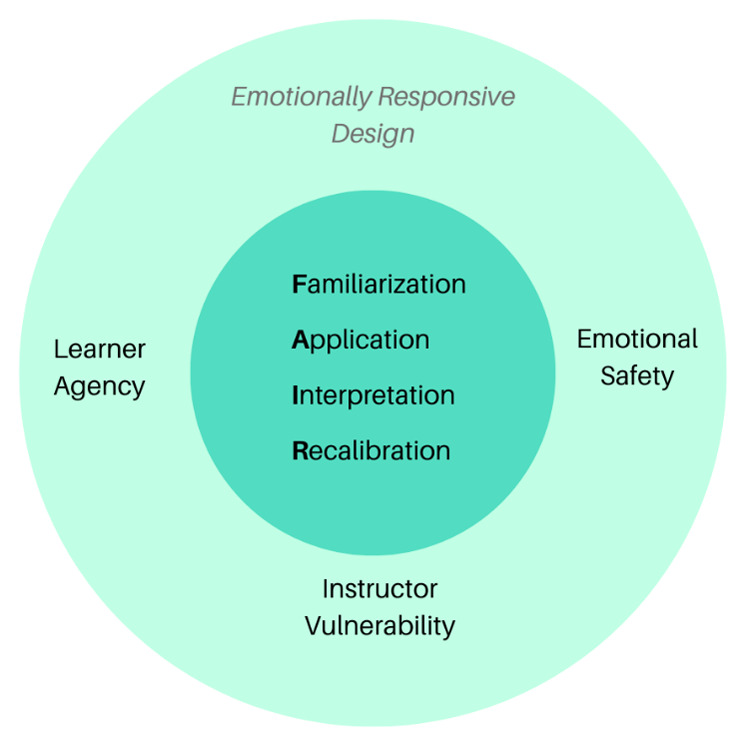
Conceptual Model.

We developed the FAIR framework to engage with the affective and ethical dimensions of GenAI integration in education. Learners progress from demystifying tools (Familiarization) to experimenting with real tools (Application), to critically reflecting on the pedagogical and ethical implications of GenAI (Interpretation), and finally, to iteratively refining their practice (Recalibration) within a community. See Figure 1. The FAIR framework was exemplified through the course assignments (See Appendix Supplementary Materials 1) and structured the learning experience as follows:

- **Familiarization**: This initial phase was designed to demystify the technology and provide low-stakes hands-on exploration. In the first two assignments, learners were guided to “explore and compare” different tools. This phase was focused on discovery rather than proficiency, allowing learners to confront their initial anxieties.
- **Application:** Once learners had a basic familiarity, the next phase provided guided opportunities to apply GenAI to their professional context. In the following assignments, learners were tasked with using GenAI to “create or redesign” a real-world educational component, such as course objectives or study guides. This step was crucial for building agency and helping learners see the immediate, practical relevance of the tools.
- **Interpretation:** This phase invited critical reflection on the broader implications of GenAI use. This phase was not just a technical step but an ethical and professional one. Learners had to “critically evaluate the considerations and potential challenges” of GenAI in their specific professional context, including identifying ethical and logistical hurdles.
- **Recalibration:** Finally, this phase emphasized iterative refinement and a commitment to future-oriented learning. Through peer feedback and structured self-reflection, learners synthesized their experiences. The “Final Reflection” assignment explicitly asked learners to analyze how their “perceptions have evolved,” identify “key takeaways,” and articulate how they “plan to keep abreast of innovations in AI,” solidifying their new identity as reflective, capable leaders.

This model worked synergistically with the emotionally responsive course design, which directly addressed the affective dimension of the learner experience.

### Emotionally Responsive Design

Core to the emotionally responsive course design were three interrelated features: emotional safety, instructor vulnerability, and learner agency [7,8,9]. We wanted to create an environment where learners could explore their responses to GenAI technologies. Instructor vulnerability, modeled through the transparent use of GenAI in assignment design and a co-learning stance, invited participants into a shared space of discovery. Finally, learner agency enabled participants to tailor their participation to their own contexts, reinforcing relevance, ownership, and intrinsic motivation. These features created a learning environment that helped learners navigate professional identity shifts prompted by AI.

### Emotional Safety

Emotional safety was foundational to normalizing the uncertainty and apprehension that learners expressed. We operationalized emotional safety asynchronously by using transparent rubrics and flexible assignment tracks to lower anxiety. We also designed for low-stakes experimentation, such as a GenAI image assignment focused on “curiosity and engagement” rather than technical skill. Most importantly, instructors provided personalized, dialogical feedback on reflective assignments. This feedback validated learners’ shared fears and anxieties, explicitly reframing them as an expected response to disruption and creating a space where learners felt gently and supportively introduced.

### Instructor Vulnerability

Instructors modeled vulnerability to dismantle the traditional expert-novice hierarchy and foster a shared co-learning stance. Vulnerability was not just a theoretical posture; it was enacted through concrete actions. For example, instructors openly disclosed their own use of GenAI in designing the course, including a formal “Declaration of AI Usage” in the syllabus stating that assignment descriptions were co-created with AI. In asynchronous discussions and feedback, instructors also shared their own challenges. They navigated the technology’s limitations alongside participants, inviting learners into a shared space of discovery and destigmatizing their own uncertainty.

### Learner Agency

Learner agency was central to ensuring the course was relevant and intrinsically motivating. We established agency primarily through assignment choice. For instance, the “Understanding AI Chat models” assignment provided two distinct tracks: one for learners “New to LLMs” and another for those “Experienced with LLMs”. This choice allowed learners to self-select the level of challenge that suited them. Furthermore, assignments explicitly required participants to use their “personally relevant materials” and “specific professional context”, aligning their GenAI experimentation with their own practice and reinforcing ownership of their learning.

Table 1 provides a quick implementation guide for adopting the FAIR framework, designed around emotionally responsive learning design. These strategies can be adopted in short hour-long training workshops and longer courses.

**Table 1:** Implementation Guide.


APPLY THE FAIR FRAMEWORK	

Familiarization	Guided, low-stakes exploration of tools. Compare 2 tools; prompt: “What surprised you? What felt uncomfortable?”

Application	Apply GenAI to real, contextual tasks. “Think of a task you need to complete as part of your job. How can you use GenAI for it?”

Interpretation	Reflect on ethical, pedagogical, and contextual implications. “Identify risks (bias, hallucinations, privacy, learner dependence) and mitigations in your context.”

Recalibration	Iterate based on peer feedback and evolving understanding. Include an identity-oriented reflection and next-step plan.

**APPLY EMOTIONALLY RESPONSIVE DESIGN**	

Establish Emotional Safety	Use transparent rubrics, low-stakes exploratory tasks, and explicit normalization of discomfort. Provide feedback that validates emotional responses and reframes uncertainty as expected. Name identity threat as expected, not as a deficiency.

Model Instructor Vulnerability	Disclose your own use of GenAI in course design, narrate your learning process, and demonstrate uncertainty openly. This vulnerability disrupts hierarchy and invites co-learning. Disclose your own GenAI use and limits; adopt a co-learning stance.

Build Learner Agency	Offer choice among assignment tracks; require learners to use materials from their own professional contexts; position them as experimenters rather than novices. Self-selected use case.

Close With Identity Reflection	Prompt learners to articulate how their perceptions shifted and how they plan to continue engaging with GenAI in their roles. “How has my role shifted, and who will I support next?”


## Outcomes of Innovation

This course has been conducted with 40 learners in three cohorts. Learners completed a pre-course survey to self-assess their prior experience with GenAI, their emotional responses to the topic (e.g., excitement, fear, apprehension), and specific learning goals. The survey was designed to surface both cognitive readiness and affective stance. Learners also completed an end-of-course reflection. Finally, participants from the first cohort (n = 12) were invited to participate in semi-structured interviews. Five of the 12 learners from the first cohort participated in interviews six months after completing the course. These data points were analyzed. This study was reviewed by our Institutional Review Board and deemed exempt.

Survey results revealed a range of prior experience with GenAI among learners. 65% (n = 26) had used GenAI tools or applications in some capacity, and only 5% (n = 2) indicated no prior knowledge. Emotional responses reflected ambivalence and complexity. Apprehension (72.5%; n = 29) and enthusiasm (67.5%; n = 27) were the most common, but over half (55%; n = 22) reported excitement. The range of affective responses reinforced the need for a psychologically safe, learner-centered learning environment.

Thematic analysis of learner comments from the survey, reflection, and interviews reinforced the importance of an emotionally responsive design approach to the structured FAIR framework. Table 2 provides the themes and exemplar quotations. Learners noted that the emotional safety that was built into the course led to confidence to explore new technologies. Hands-on use of various tools strengthened their familiarity with them, and giving learners agency to choose how they applied the tools helped them see the immediate applicability of the technology. The space for reflection enabled interpretation, which led to recalibration.

**Table 2:** Themes and Quotations.


THEME	EXEMPLAR QUOTATION

Emotional safety	“Now, having been gently and supportively introduced into this world… I feel like I have something of a foothold to explore.”

Hands-on experience and Learner choice of tasks	“I used ChatGPT to create a simulation we’re running next week… This was immediately useful.”

Reflection	“Reflecting on these experiences allowed me to better understand the concerns associated with bias, hallucinations, legal implications”

Recalibration	“I aim to refine the GPT further and study its application in various health professions settings.”

Moving from apprehension to confidence	“I started off afraid and felt like I didn’t belong here… now I’m eager to explore more.”

Helpful tool	“I now have an AI partner in curriculum design—even when no one else is available.”

Awareness of ethical tensions	“I still feel conflicted” and “I worry about the critical thinking skills of students who lean too heavily on AI.”

Continued use	“I’ve joined an AI work group… we’re trying to think about how we implement this across clinical, academic, and administrative roles.”

Empowering peers	“As an educator and voice in medical ethics, I feel a responsibility to help others navigate AI thoughtfully.” “I shared it with my colleagues, and they were like, ‘This is awesome.’ We use it now to summarize long emails, draft lesson plans. We’ve started teaching others how to use it.”

Leading change	“I now feel responsible to lead change—not just keep up with it.”


Designing a course using this approach revealed that learners experienced a shift in their perception of AI, moving from apprehension to confidence. They realized GenAI could be a helpful tool, yet they demonstrated a critical awareness of the persistent ethical tensions in using GenAI in healthcare environments. Learners reported continued use and integration of generative AI tools across various educational and administrative domains. Beyond personal use, the learners were also committed to sharing this information with colleagues, empowering them, and leading change at an organizational level.

Taken together, these outcomes support our thesis that emotional readiness is the central determinant of GenAI adoption, not a secondary concern. Learners’ shifts from fear and self-doubt to curiosity, belonging, and leadership align with our claim that an emotionally responsive environment fosters the psychological safety needed to engage with a disruptive topic. The FAIR framework functions as the scaffolding that channels that safety into action, moving participants from demystification to authentic application, critical interpretation, and iterative recalibration. With this approach, GenAI becomes something learners can critically test, adapt, and teach within their own contexts rather than something to avoid or passively consume.

## Critical Reflection

Responses from our learners suggest that the emotionally responsive design and FAIR framework led to durable shifts in mindset, behavior, and institutional engagement. While many professional development models emphasize tool use and ethical compliance, our findings suggest that emotional safety, identity affirmation, and opportunities to connect with peers are important for effective and sustained engagement with GenAI. The shift in the learners’ response to AI from apprehension to confidence and resistance to leadership reveals that when emotional barriers are addressed directly, learners do not simply gain competence; they reimagine their professional roles and responsibilities.

This strategy has broad implications for the adoption of GenAI in health professions education. If educators feel unprepared or uncertain of their place in an AI-mediated future, integration efforts will likely stall. In contrast, emotionally responsive pedagogy grounded in the FAIR framework positions educators not just as users of tools, but as co-creators of a changing educational paradigm. This design is also adaptable. The FAIR framework and its core values of emotional safety and learner agency can be embedded in courses, faculty development series, or brief workshops. By addressing core affective needs, FAIR provides a scalable and flexible model for GenAI education.

While our process proved successful in these areas, our critical reflection also identified pitfalls in the design and implementation that others should consider.

First, an undeniable pitfall of this process is its high resource intensity. Operationalizing emotional responsiveness asynchronously by providing personalized, validating, and non-evaluative feedback on reflections is time-consuming. Modeling vulnerability and managing learners’ anxieties also requires faculty to perform emotional labor. These limitations present a challenge for scalability. Some ways to address scala bility are to start by incorporating high-touch facilitation at a few points (e.g., early normalization of discomfort, feedback on the first authentic application artifact, and end-of-course recalibration). For other elements, reusable templates such as templated feedback banks, rubric-aligned audio/video responses, and structured peer-feedback protocols can be used. Programs can also scale through co-facilitation models (faculty champions or trained alumni as near-peers), cohort caps matched to facilitator capacity, and “bring-your-own-artifact” workflows that anchor work in existing teaching materials and reduce preparation load.

Second, a key challenge in refining our process is that our two core innovations of the emotionally responsive design and the structured FAIR framework are deeply entangled. A pitfall of this design is that it is difficult to isolate which component is doing the heavy lifting. We cannot definitively prove if the logical FAIR framework would have been less successful without the emotional scaffolding, or if the emotional support would have felt hollow without the structured FAIR assignments.

However, we argue that this entanglement is the innovation. Our thesis is that a logical framework (FAIR) is necessary for structure, but it is insufficient for a disruptive topic like GenAI. The emotionally responsive design is the necessary precondition that creates a safe environment for learners to engage with the framework. The qualitative data, in which learners explicitly credit the “gently and supportively introduced” nature of the course for their shift from “afraid” to “eager”, provide strong evidence for this co-dependent relationship.

We recommend integrating emotionally responsive design and the FAIR framework as a single coupled intervention rather than as separable “content” and “climate” features. Each FAIR phase should include at least one safety-and-agency mechanism such as low-stakes exploration, normalized uncertainty, or values-based reflection. In lower-resource settings, a minimum implementation could consist of brief demystification, one authentic application artifact, a guided interpretation step on local risks/mitigations, and a required recalibration revision informed by structured peer or facilitator feedback.

## Limitations and Next Steps

This study is limited by its small sample size (n = 40) and reliance on self-reported data from course reflections and surveys. Participants were drawn from a voluntary, self-selected group of educators, which may introduce bias toward those already interested or motivated to engage with GenAI. Additionally, though our longitudinal data does show organizational impact, our sample size is small (n = 5). However, we caution against interpreting the low interview participation as evidence of low enthusiasm. Conducting longitudinal follow-ups with busy healthcare professionals is challenging, and attrition was often due to external factors such as clinical schedules or administrative burdens. The primary value of our follow-up data is in demonstrating proof of concept for sustained change. The rich data from these five learners who reported “continued use”, institutional dissemination, and teaching their peers, provides significant evidence that the course’s impact can be durable, directly supporting the goal of our innovation.

We continue to collect data from subsequent course cohorts, focusing on longitudinal outcomes and institutional dissemination. Future work will examine how participants apply GenAI in their teaching over time, including changes in curricular design, faculty development initiatives, and peer mentoring practices. We also aim to explore the transferability of the FAIR framework across different professional development contexts, including interprofessional and clinical team settings. Additionally, we plan to develop a toolkit for emotionally responsive GenAI education that can be adapted by faculty developers and continuing professional development leaders. Ultimately, our goal is to build an evidence-informed model for GenAI adoption that centers not only on tool proficiency but also on emotional readiness, professional identity, and ethical leadership.

## Conclusion

The FAIR framework was embedded within the learning environment, specifically designed to foster emotional safety and normalize uncertainty. This design treated emotional engagement as a necessary foundation for pedagogical innovation with GenAI. Learners’ fear and anxiety were not seen as deficits, but as expected responses to disruption. Our goal was not only to build GenAI fluency, but also to develop reflective, future-ready educators equipped to navigate the shifts GenAI brings to the health professions.

## Additional File

The additional file for this article can be found as follows:

10.5334/pme.2280.s1 Appendix Supplementary Materials 1. HPE 615: AI in Health Professions Education: Implications for educators and leaders.
