# Supplementary material for: It’s Not Just About the Tools: Emotionally Responsive GenAI Education
Source: Perspect Med Educ. 2026 Apr 8;15(1):356–62. doi: 10.5334/pme.2280 (PMC13068100; doi:10.5334/pme.2280)
Supplement: Appendix Supplementary Materials 1. — HPE 615: AI in Health Professions Education: Implications for educators and leaders. [file pme-15-1-2280-s1.pdf]

# HPE 615: AI in Health Professions Education: Implications for educators and leaders

**DECLARATION OF AI USAGE**

Please be aware that assignment descriptions in this course have been generated using artificial intelligence (AI) tools and subsequently reviewed and approved by the instructor. This approach aims to enhance clarity and ensure alignment with course objectives. If you have any questions or require further clarification regarding assignment details, feel free to reach out.

## Assignment Descriptions

### Pre-Course Survey

| | |
|---|---|
| **Purpose of the Assignment** | To gauge students' baseline knowledge, motivations, and concerns regarding AI in health professions education. |

**Due Date: 1/7**

Complete the following survey:

### Understanding AI Chat models

| | |
|---|---|
| **Purpose of the Assignment** | As technology becomes more integrated into healthcare, it's crucial for health professionals to be familiar with and understand the capabilities of tools like AI. Incorporating them into education prepares students for future technological advancements in the field.<br><br>This assignment helps you deepen your understanding of Large Language Models (LLMs) by tailoring activities to your current experience level. Whether you're new to using AI tools or an experienced user, you'll engage with LLMs in a way that expands your skills and understanding. |

**Choose Your Path:**

Please select the assignment track that best matches your familiarity with AI tools and prompt crafting. If you're unsure which track to choose, feel free to reach out for guidance!

**Track 1: For Learners New to LLMs**

**Objective:** Explore and compare three different AI models (e.g., ChatGPT, Gemini, Claude, Perplexity) to understand their features and potential applications.

**Instructions:**

1. Setup & Exploration:
   - Register or access the respective platforms that host ChatGPT, Gemini, and Claude (or another platform you want to try).
   - Familiarize yourself with the basic functions of each chatbot by engaging in general conversations with them.
   - Get Insights: Ask each AI model simple, unrelated questions (e.g., general trivia) to explore how they respond and any quirks in their outputs.

2. Scenario Creation:
    - Design two different HPE-related scenarios that you'd like to explore with each chatbot. These could include:
        - Answering a complex medical question.
        - Discussing the ethics of a particular medical procedure.
        - Seeking clarification on medical terminology or concepts.
3. Interaction & Documentation:
    - Engage each chatbot using the scenarios you've designed.
    - Document each chatbot's responses to the scenarios. Copy the prompt/s and responses of each tool and include it in your document.
    - Use a table to compare outputs clearly. For example:

| Scenario | ChatGPT | Gemini | Claude |
|---|---|---|---|
| **Scenario 1:** | [Response] | [Response] | [Response] |
| **Scenario 2:** | [Response] | [Response] | [Response] |

4. Analysis & Reflection:
    - Compare and contrast the responses from the LLMs for each scenario.
        - **Depth of Answers:** How detailed and comprehensive are their explanations?
        - **Nuance and Clarity:** Do they offer thoughtful, clear, and well-organized responses?
        - **Accuracy:** Are the facts correct? Identify any discrepancies.
    - Reflect on the potential uses of these chatbots in medical education. Consider their strengths, limitations, and areas of improvement.
    - Capture nuances, depth of answers, accuracy, and any other relevant observations.

### Track 2: For Learners Experienced with LLMs

**Objective**: Apply your existing knowledge of LLMs by creating a custom GPT using OpenAI's fine-tuning capabilities or exploring advanced prompt engineering.

1. **Choose a Focus**:
    - Select a specific task relevant to healthcare education, such as:
        - Customizing a GPT to generate tailored quiz questions.
        - Developing prompts to simulate patient scenarios.
2. **Plan & Execute**:
    - If using fine-tuning, curate a small dataset (5–10 examples) to train the GPT.
    - If focusing on advanced prompts, experiment with prompt chains or parameter tuning to achieve refined outputs.
3. **Document Your Process**:
    - Describe the steps you took, including any challenges faced and solutions applied.
    - Provide sample outputs demonstrating the performance of your custom GPT or advanced prompts.
4. **Reflect**:
    - How did creating or customizing a GPT deepen your understanding of LLMs?
    - What potential applications do you see for your work?

**Deliverables**:

- A document including:
    - A description of your focus and approach.
    - Sample outputs of your custom GPT or advanced prompts.

- Reflections on the process and potential applications.

**Grading Rubric:**
Your participation and posts will be evaluated based on:

| | **Exemplary**<br>This represents the highest level of mastery. Learners not only meet but often exceed the expected standards. Their performance serves as a model for others. | **Proficient**<br>Learners consistently meet the expected standards. While there's room for growth and enhancement, their performance is satisfactory and competent. | **Basic**<br>This level indicates that performance is below the expected standard. Additional training, guidance, or resources might be required. |
|---|---|---|---|
| **Depth of Exploration:** How thoroughly you've engaged with and explored each chatbot. (25) | | | |
| **Analysis Quality:** Depth, clarity, and insightfulness of your comparative analysis. (25) | | | |
| **Report Presentation:** Structure, coherence, and presentation of your report. (25) | | | |
| **Reflection:** Thoughtfulness in considering the potential applications and implications of using these chatbots in medical education. (25) | | | |

## Exploring AI-Generated Images: A Creative Journey

**Purpose:**
This assignment is designed to help you confidently explore and engage with AI image generation tools. By experimenting with these tools, you'll develop a better understanding of their creative possibilities and learn how AI interprets and responds to your prompts.

**Instructions: Let's Get Started!**

### *Step 1: Imagining Your Creation*

1. Take a moment to think of an image that excites you—perhaps something you've always wanted to visualize.
2. Jot down your ideas. What colors, themes, or elements do you want in the image? If you're unsure, consider something simple to start with.

### *Step 2: Creating Your Image*

1. Open an AI image generation tool of your choice (e.g., DALL-E, MidJourney, or another tool you are comfortable with).
2. Input your initial idea into the tool as a prompt.
3. Experiment with variations! Change small details in your prompts or settings to see how they influence the outcome. For example, tweak the style, colors, or details.

### *Step 3: Reflection and Understanding*

After creating at least three versions of your image:

1. Compare them. How do they differ? How closely do they match your original idea?
2. Reflect on your creative journey:
    - Were there moments that surprised you?
    - Did the AI bring any unexpected interpretations to your prompts?
    - How did this process differ from traditional art creation?

**Deliverables: Share Your Experience**
Submit a document that includes:

1. **Your three (or more) AI-generated images.** Make sure each image is labeled with the exact prompt you used.
2. **Your reflection:** Answer the questions in Step 3, sharing your thoughts and insights about the process.

**How You'll Be Assessed:**

1. **Creativity & Originality**: How well you explored and used the tool's capabilities.
2. **Thoughtfulness in Reflection**: Depth and honesty in your analysis of the images and creative process.
3. **Effort**: Demonstration of your willingness to experiment and engage.

---

**We're Here to Help!**

If you encounter any challenges or feel unsure about the process, please don't hesitate to reach out. This is a learning journey, and your curiosity and engagement matter most.

## Grading Rubric

| Category | Criteria | Points |
|---|---|---|
| **Creativity and Originality** | - Exceptional creativity; unique or complex ideas in prompts; thoughtful experimentation. (25–30 points) | **30 points** |
| | - Moderate creativity; conventional ideas with limited scope. (15–24 points) | |
| | - Minimal creativity or effort; basic or repetitive prompts and images. (0–14 points) | |
| **Experimentation and Use of Tool** | - Extensive exploration; varied prompts/settings; clear effort to understand tool. (20–25 points) | **25 points** |
| | - Limited variation in prompts/settings; moderate effort in analyzing results. (10–19 points) | |
| | - Minimal exploration; little to no variation in prompts or settings. (0–9 points) | |
| **Adherence to Prompts** | - Images closely align with prompts; clear connection between prompt and output. (13–15 points) | **15 points** |
| | - Images mostly align with prompts; minor inconsistencies. (8–12 points) | |
| | - Little alignment between prompts and generated images. (0–7 points) | |
| **Reflection and Insight** | - Insightful, thoughtful, and detailed analysis of challenges, surprises, and AI process. (16–20 points) | **20 points** |
| | - Clear reflection; addresses main points but lacks depth/detail. (10–15 points) | |
| | - Vague, superficial, or missing key aspects of the creative process. (0–9 points) | |
| **Presentation and Organization** | - Well-organized, visually appealing; images properly labeled; reflections structured. (8–10 points) | **10 points** |
| | - Mostly organized and easy to follow; minor presentation issues. (5–7 points) | |
| | - Poorly organized or incomplete deliverables. (0–4 points) | |

## Using Generative AI in Medical Education Design

| **Purpose of the Assignment** | To leverage generative AI in creating or redesigning an educational component—be it course objectives, assignments, or other learning materials—in medical education.<br><br>This assignment encourages you to think critically about the integration of AI in educational design. While generative AI can be a powerful tool, always consider the needs of learners and the goals of the curriculum when crafting or refining materials. |
|---|---|

**Instruction:**

1. Selection of Educational Component:
   - Choose an educational component you'd like to create or redesign. This could be anything including:
     - Course objectives for a new topic.
     - Assignments or assessment tasks.
     - Study guides or supplementary learning materials.
2. Select the Generative AI tool:
   - You can select any LLM and other tools you like. Or a combination of them!
3. Utilize Generative AI:
   - Use the generative AI platform/tool to assist you in creating or redesigning your chosen educational component. This could involve:
     - Generating potential course objectives.
     - Drafting assignment prompts or questions.
     - Creating summaries, flashcards, or other learning aids.
   - Once you've generated content, refine it. Remember, while AI can produce valuable outputs, it often requires a human touch for context, relevance, and clarity.
4. Documentation & Reflection:
   - Compile a document that includes:
     - The original version (if it's a redesign).
     - The AI-generated version.
     - Your refined and final version.
   - Reflect on the process:
     - How did generative AI aid in the creation/redesign?
     - What challenges did you face?
     - In what scenarios might you consider using generative AI in the future for educational design?

**Grading Rubric:**

Your participation and posts will be evaluated based on:

| | **Exemplary** | **Proficient** | **Basic** |
|---|---|---|---|
| **Creativity & Innovation** (40) | Demonstrates a high degree of originality and innovative use of generative AI. | Shows creativity and some innovative use of AI, but may be somewhat conventional in places. | Uses AI in basic ways; limited creativity in the educational component. |
| **Refinement & Personal Touch** (40) | Content is thoroughly refined, demonstrating deep understanding and context. Personal touch is evident throughout. | Content shows good refinement, with areas of personal touch. Some minor areas may lack depth. | Content is somewhat refined, but lacks consistent depth or personal touch in places. |
| **Reflection** (20) | Reflection is deep, insightful, and provides a comprehensive understanding of the AI's role in the design process. | Reflection is thoughtful with some insights about AI's role, but might lack depth in places. | Basic reflection on the process with limited insights or depth. |
| | | | |

## Leadership in Implementing AI

| | |
|---|---|
| **Purpose of the Assignment** | To critically evaluate the considerations and potential challenges of implementing AI in your specific professional context, and to strategize solutions and leadership approaches to address those challenges.<br><br>This assignment is an opportunity for introspection and foresight. While technological advancements like AI can greatly enhance processes and outcomes, thoughtful and strategic leadership is crucial to navigate the associated complexities. Reflect on both the micro and macro aspects of AI implementation and envision yourself as a pivotal leader in this transformative journey. |

**Instructions:**

1. Contextual Considerations:
   - Begin by briefly describing your professional context
   - Enumerate the specific considerations you, as a leader, would need to keep in mind when introducing AI into this setting. Consider factors such as the organization's mission, stakeholders' needs, existing technological infrastructure, and the nature of services/products offered.
2. Identification of Potential Challenges:
   - Based on your context, identify and elaborate on at least three major challenges you anticipate facing when implementing AI. These challenges can be technical, ethical, logistical, financial, or related to human resources.
3. Addressing Challenges:
   - For each identified challenge:
     a. Propose a strategic solution or approach to mitigate or overcome the challenge.
     b. Describe the leadership skills and strategies you would employ to guide your team or organization through the challenge.
     c. Reflect on any resources, collaborations, or external support you might need to successfully address the challenge.
4. Conclusion:
   - Summarize your findings and reflect on the broader implications of AI implementation in your field. Discuss the balance between staying ahead technologically and ensuring ethical, effective service or product delivery.

**Grading Rubric:**
Your participation and posts will be evaluated based on:

| |
|---|
| **Contextual Insight:** Demonstrated understanding of your specific professional context and its unique considerations. (20) |
| **Depth of Challenge Analysis:** Comprehensive identification and elaboration of potential challenges. (20) |
| **Solution Strategy:** Practicality, feasibility, and foresight in proposed solutions to address challenges. (25) |
| **Leadership Approach**: Clarity and thoughtfulness in described leadership strategies and skills. (15) |
| **Overall Cohesion**: Logical flow, clarity, and coherence of the entire document. (10) |

## Final Reflection

| | |
|---|---|
| **Purpose of the Assignment** | To introspectively analyze how the course has impacted your understanding and perception of AI, gauge your newfound motivations, and identify the key takeaways that resonated the most with you.<br>This assignment encourages deep introspection and personal engagement with the course content. Consider it an opportunity to consolidate your learning, recognize its influence on your |

| | |
|---|---|
| | worldview, and chart a path forward in your AI journey. |

**Instructions:**

1. Changed Perceptions:
   - Begin your reflection by describing your initial perceptions or beliefs about AI prior to starting this course.
   - Discuss how and in what ways these perceptions have evolved or changed throughout the course.
2. Newfound Motivations:
   - Reflect on how this course has influenced your future endeavors concerning AI. Are there specific areas within AI you are now motivated to explore further, or career paths you're considering?
   - Discuss any immediate actions or steps you plan to take as a result of this motivation.
3. Surprises and Revelations:
   - Were there any topics or pieces of information that took you by surprise or challenged your existing beliefs? What were they and why were they surprising to you?
   - Reflect on why these surprises were significant in your learning journey.
4. Key Takeaways:
   - Identify and elaborate on at least three key takeaways from the course. These could be insights, skills, pieces of knowledge, or overarching themes.
   - Discuss why these takeaways are significant for you and how you envision applying or integrating them into your professional or personal endeavors.
   - Thinking about the way that material was taught during the course or course assignments, what was most impactful to your learning the course content?
   - What methods were least helpful to you?
   - Was there anything unique or different about the ways that the content in this course was delivered? What was that?
   - What impact, if any, did group or peer learning have on your learning about the course content?
   - How do you plan to keep abreast of innovations in AI as you move forward?

**Grading Rubric:**

Your participation and posts will be evaluated based on:

| | **Exemplary** | **Proficient** | **Basic** |
|---|---|---|---|
| **Depth of Reflection:** Demonstrated introspection and depth in your reflections, showing genuine engagement with the course content. (20) | | | |
| **Clarity and Cohesion:** Logical flow of thoughts, clear expression of ideas, and overall coherence in your reflections. (20) | | | |
| **Solution Strategy:** Practicality, feasibility, and foresight in proposed solutions to address challenges. (20) | | | |
| **Specificity:** Providing specific examples, modules, or moments from the course that influenced your perceptions and decisions. (20) | | | |
| **Future Implications:** Clear articulation of how the course will influence your future actions, decisions, or career paths concerning AI. (20) | | | |
